# Patterns and change rates of glacial lake water levels across High Mountain Asia

**DOI:** 10.1093/nsr/nwaf041

**Published:** 2025-02-11

**Authors:** Yingzheng Wang, Donghai Zheng, Guoqing Zhang, Jonathan L Carrivick, Tobias Bolch, Weiwei Ren, Lei Guo, Jianbin Su, Shiwei Yuan, Xin Li

**Affiliations:** National Tibetan Plateau Data Center (TPDC), State Key Laboratory of Tibetan Plateau Earth System, Environment and Resources (TPESER), Institute of Tibetan Plateau Research, Chinese Academy of Sciences, China; College of Earth and Environmental Sciences, Lanzhou University, China; National Tibetan Plateau Data Center (TPDC), State Key Laboratory of Tibetan Plateau Earth System, Environment and Resources (TPESER), Institute of Tibetan Plateau Research, Chinese Academy of Sciences, China; National Tibetan Plateau Data Center (TPDC), State Key Laboratory of Tibetan Plateau Earth System, Environment and Resources (TPESER), Institute of Tibetan Plateau Research, Chinese Academy of Sciences, China; School of Geography and water@leeds, University of Leeds, UK; Institute of Geodesy, Graz University of Technology, Austria; National Tibetan Plateau Data Center (TPDC), State Key Laboratory of Tibetan Plateau Earth System, Environment and Resources (TPESER), Institute of Tibetan Plateau Research, Chinese Academy of Sciences, China; School of Geosciences and Info-physics, Central South University, China; National Tibetan Plateau Data Center (TPDC), State Key Laboratory of Tibetan Plateau Earth System, Environment and Resources (TPESER), Institute of Tibetan Plateau Research, Chinese Academy of Sciences, China; National Tibetan Plateau Data Center (TPDC), State Key Laboratory of Tibetan Plateau Earth System, Environment and Resources (TPESER), Institute of Tibetan Plateau Research, Chinese Academy of Sciences, China; National Tibetan Plateau Data Center (TPDC), State Key Laboratory of Tibetan Plateau Earth System, Environment and Resources (TPESER), Institute of Tibetan Plateau Research, Chinese Academy of Sciences, China

## Abstract

Using satellite altimetry data, this work quantifies the inter-annual trends and intra-annual fluctuations in water levels of glacial lakes in High Mountain Asia during 2019-2023.

Glacial lakes, which form and enlarge due to glacier retreat and meltwater accumulation, serve as indicators of climate change and glacier mass loss [1]. High Mountain Asia (HMA), which encompasses the Tibetan Plateau, greater Himalaya and Tien Shan, is recognized because its glacier coverage is the largest outside of the Antarctic and Arctic [2]. Atmospheric warming across HMA has occurred at a rate that is twice that of the global average [3] and has led to accelerated glacier mass loss and a substantial increase in the number and area of glacial lakes [4]. The concentrated population and infrastructure development, including hydropower and transport networks, in the downstream areas of HMA make it the most vulnerable region to glacial lake outburst floods (GLOFs) in the world [5].

The occurrence of a GLOF is often influenced by multiple factors related to the glacier, the glacial lake itself, the surrounding environment and the structural integrity of the lake dam. Notably, fluctuations in lake water levels can pose a significant threat to the stability of the lake dam [6]. Changes in glacial lake water levels (GLWLs), whether long-term (ranging from several days, weeks, months, years or even longer) or short-term (instantaneous changes), are fundamentally influenced by the difference between the water inflow and outflow. For the long term, if the static water pressure exceeds the threshold of a dam, then it can lead to ruptures in the internal or surface pipes of

the dam, resulting in GLOFs. In the short term, sudden water-level increases, often caused by unexpected disturbances such as snow/ice/rock avalanches or intense precipitation, can generate rolling surges that may surpass the lowest crest of the natural dam and/or damage its structure, thereby triggering GLOFs [5,7,8]. Enhanced measurement of GLWLs is crucial for assessing changes in lake volumes, understanding GLOF flooding and managing hazards and water resources.

Remote sensing technology offers great opportunities for monitoring changes in glacial lakes. While *in*  *situ* measurements provide detailed validation data, they are labor- and resource-intensive, and spatially limited to a few spot measurements [9]. Many studies have examined the spatial and temporal characteristics of glacial lake extent at decadal [10], quinquennial [1] and even annual scales [11]. However, GLWLs across the entire HMA have not been thoroughly considered, particularly due to the high number (25 385) and extensive coverage of lakes (1 746.49 km²) [12], as well as the predominance of small lakes with narrow bounding rectangles in complex mountainous terrain [13] (Fig. S1). In this study, we provide a solution to these issues by applying a periodic fluctuation model to glacial lake-level retrievals from ICESat-2 laser altimetry and Sentinel-3 radar altimetry data between 2019 and 2023 across HMA. That enables us to reveal spatial patterns, interannual change

rates and intra-annual amplitudes, and to interpret the environmental controls on these. Given the small size and narrow shape of glacial lakes and their intra-annual and interannual water-level variations, this study differentiates itself from existing research on interannual water-level variations in large reservoirs or lakes. Furthermore, based on measurements of all glacial lakes, we conduct a quantitative analysis of the factors that influence water-level variations, distinguishing it from traditional qualitative discussions or regional correlation analyses of water-level factors in lakes or reservoirs.

We obtained mean and median values of –0.00 ± 0.02 and 0.00 ± 0.01 m a^−1^, respectively, for the interannual change rates in the levels of 442 glacial lakes between 2019 and 2023 (Fig. 1). These findings suggest that, overall, glacial lake levels of HMA are in dynamic equilibrium. However, this region-wide calculation hides spatial–temporal variability. We find that 225 lakes (50.90%) exhibit rising levels, with mean and median values of 0.09 ± 0.03 and 0.05 ± 0.01 m a^−1^, respectively, whilst 217 lakes (49.10%) have persistently lowering levels, with mean and median values of –0.10 ± 0.03 and –0.07 ± 0.01 m a^−1^, respectively. Figure 1 illustrates that levels tend to be rising in the Central Himalaya, Eastern Himalaya, North-Western Tien Shan and Nyainqêntanglha, with the largest increase in the Northern-Western Tien Shan (mean: 0.04 ± 0.04 m a^−1^, median: 0.04 ± 0.03 m a^−1^). In contrast, glacial lakes in regions including the Dzhungarian Alatau, Eastern Hindu Kush, Eastern Kunlun Shan, Eastern Tibetan Mountains, Eastern Tien Shan and Gangdise Mountains are experiencing lowering levels, with the largest decrease in the Gangdise Mountains (mean: –0.09 ± 0.06 m a^−1^, median: –0.04 ± 0.03 m a^−1^). Of the 364 (82.35%) open glacial lakes, more than half (206) are experiencing lowering levels (Fig. S5a). In contrast, of the 78 closed glacial lakes, 67 (85.90%) have rising levels (Fig. S5b). The change rate of lowering levels in open lakes is likely due to their natural drainage over unconsolidated lake dams, facilitating water outflow through erosion. Conversely, closed lakes, without natural drainage outlets, experience rising levels due to water accumulation over time. So, the statistical patterns (Fig. S5a and b) observed in our study are consistent with the expected general behaviors of these closed glacial lakes. GLWLs and glacier area are correlated (Fig. S5c) with a positive linear relationship (*K *= 0.002, *R* = 0.095, *P* = 0.045). Due to the limited number of 442 glacial lakes measured in this study, the correlation is relatively weak. Nevertheless, the relationship passed the significance test, indicating statistical significance. Furthermore, this relationship suggests that larger glacier areas in the watershed of each glacial lake, which typically imply greater mass loss during 2019–23 and thus produce more meltwater, are associated with rising lake levels, and this is also consistent with the physical principles. Overall, glacial lakes that are open and have relatively small glacier areas in their watersheds tend to experience a decrease in interannual water levels (Fig. S5a).

**Figure 1.**
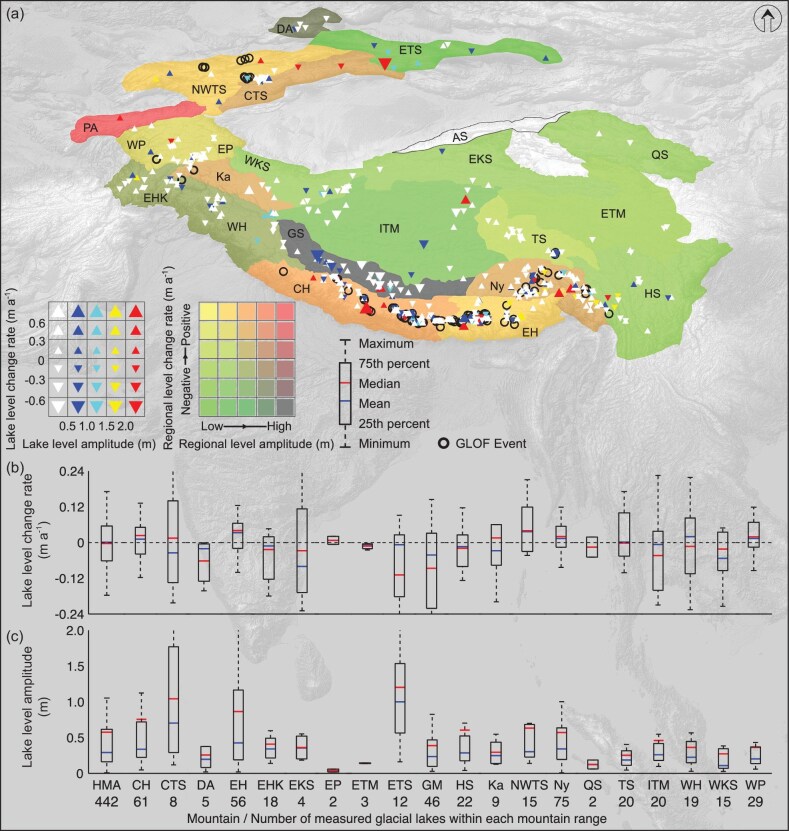
Interannual change rates and intra-annual amplitudes of GLWLs within HMA. (a) Upper/lower triangles represent a rising/lowering in the interannual change rates of GLWLs. Sites of previous GLOF events are based on published research provided in Table S1. Intra-annual amplitudes and interannual change rates of GLWLs are classified into five and six categories, respectively, based on mean amplitudes (for intra-annual) and mean change rates (for interannual) using natural breaks. (b) and (c) Interannual change rates and intra-annual amplitudes in GLWLs, respectively, discriminated for each mountain range.

Intra-annual amplitudes of GLWLs have a mean and median of 0.58 ± 0.06 and 0.29 ± 0.02 m, respectively (Fig. 1). Specifically, 307 glacial lakes (69.46%) show level fluctuations of <0.5 m, 72 lake levels (16.29%) fluctuate between 0.5 and 1.0 m, and the remaining 63 lakes (14.25%) experience level fluctuations of >1.0 m. Spatially, glacial lakes in the Central Himalaya, Central Tien Shan, Eastern Himalaya, Eastern Tien Shan, Northern-Western Tien Shan and Nyainqêntanglha exhibit the greatest intra-annual amplitudes. Among these, the Eastern Tien Shan shows the most pronounced fluctuations in both mean (1.20 ± 0.16 m) and median (1.00 ± 0.23 m). A median regional level amplitude of 0.34 ± 0.05 m is modeled for Nyainqêntanglha and the magnitude of that accords with *in situ* measurements (∼0.4 m between 2014 and 2021) reported for the Guangxie Co located in this area [9]. A linear positive correlation is evident between the glacial lake-level amplitude and the intra-annual precipitation amplitude (*K *= 0.003, *R *= 0.193, *P *< 0.0001) (Fig. S5d) as well as between the level amplitude and the surrounding terrain slope (*K *= 0.031, *R *= 0.289, *P *< 0.0001) (Fig. S5e). These correlations imply that glacial lakes in subregions that are characterized by high precipitation fluctuation and steeper terrain are more prone to experiencing substantial level fluctuations.

These interannual change rates and intra-annual amplitudes highlight the spatial heterogeneity in glacial lake-level dynamics across HMA. Particularly noteworthy are the Eastern Himalaya, Central Himalaya, Nyainqêntanglha and Northern-Western Tien Shan, where these patterns are most pronounced (Fig. 1). Interestingly, there is a correspondence of GLOFs that have predominantly occurred in the Central Himalaya (46 occurrences), Eastern Himalaya (43 occurrences), Northern-Western Tien Shan (31 occurrences), and Nyainqêntanglha (13 occurrences) (Fig. 1). Since GLOFs are driven by a complex interplay of internal and external factors [14] that lead to varied breach mechanisms [7] that include two types: a gradual increase in water level, leading to enhanced hydrostatic pressure that breaches the dam [15], and a rapid surge in water level that generates a wave, overtopping or damaging the dam [8], then monitoring of GLWLs is essential for assessing the potential hazard of glacial lakes and for refining the understanding of GLOF mechanisms.

This study utilizes the capabilities of the ICESat-2 laser satellite with moderate resolution and the Sentinel-3 altimetry satellites with frequent revisits, along with a periodic fluctuation model, to produce the first holistic quantification of GLWLs across HMA. The quantification of spatial patterns and interannual change rates permit the environmental controls to be elucidated. Specifically, the interannual change rate in GLWLs is influenced by the status of the lakes and the meltwater of the parent glacier. Intra-annual amplitudes of GLWLs are controlled by precipitation and terrain slope. Furthermore, our findings indicate that the Central Himalaya, Eastern Himalaya, Northern-Western Tien Shan and Nyainqêntanglha are the mountain ranges within which the most pronounced variations in GLWLs persist and those that have more frequent GLOFs.

The framework for measuring GLWLs can be applied to other global high-mountain regions, integrating additional altimetry data, including the recently launched SWOT satellite [16]. It enables the calculation of lake levels and storage changes by interpolating water levels, facilitating the quantification of how glacier meltwater affects lake volumes and aiding in the measurement of lake levels relative to dams, which supports GLOF prevention efforts. By measuring lake-level changes before and after a GLOF event, flood discharge volumes can be quantitatively assessed and initial parameters provided for GLOF simulation models. Continuous water-level monitoring helps in understanding long- and short-term variations, developing management strategies and mitigating flood risks to protect people and infrastructure.

## Supplementary Material

nwaf041_Supplemental_File

## Data Availability

For the sources and processing principles of all data used in this study, please refer to the Supplementary information.
