# Supplementary material for: Patterns and change rates of glacial lake water levels across High Mountain Asia
Source: Natl Sci Rev. 2025 Feb 11;12(3):nwaf041. doi: 10.1093/nsr/nwaf041 (PMC11881683; doi:10.1093/nsr/nwaf041)
Supplement: nwaf041_Supplemental_File [file nwaf041_supplemental_file.docx]

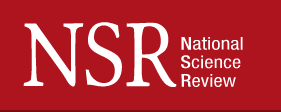


**Supplementary Information for**

**Patterns and change rates of glacial lake water levels across High Mountain Asia**

Yingzheng Wang^a,b^, Donghai Zheng^a,*^, Guoqing Zhang^a^, Jonathan L. Carrivick^c^, Tobias Bolch^d^, Weiwei Ren^a^, Lei Guo^e^, Jianbin Su^a^, Shiwei Yuan^a^, and Xin Li^a,*^

^a^ National Tibetan Plateau Data Center (TPDC), State Key Laboratory of Tibetan Plateau Earth System Science, Environment and Resources (TPESER), Institute of Tibetan Plateau Research, Chinese Academy of Sciences, Beijing, China.

^b^ College of Earth and Environmental Sciences, Lanzhou University, Lanzhou, China.

^c^ School of Geography and water@leeds, University of Leeds, Leeds, UK.

^d^ Institute of Geodesy, Graz University of Technology, Graz, Austria.

^e^ School of Geo-sciences and Info-physics, Central South University, Changsha, China.

^*^Correspondences. Email: [xinli@itpcas.ac.cn](mailto:xinli@itpcas.ac.cn) (Xin Li); [zhengd@itpcas.ac.cn](mailto:zhengd@itpcas.ac.cn) (Donghai Zheng).

# This PDF file includes:

Supplementary Texts

Supplementary Methods

Data availability

Supplementary Figures

Supplementary Table

Supplementary References

# Supplementary Texts

**Altimetry Data**

For assessing water level changes in glacial lakes across HMA region, this study utilizes altimetry data obtained from the ICESat-2 satellite and Sentinel-3A/B satellites.

ICESat-2 (Ice, Cloud, and Land Elevation Satellite-2), a cornerstone of NASA's Earth Observing System, is engineered to deliver precise measurements across diverse terrains. Launched on 15 September 2018, ICESat-2 orbits near-polar regions, completing a full Earth survey every 91 days [1]. Its hallmark, the Advanced Topographic Laser Altimeter System (ATLAS), measures surface distances with unparalleled precision using a photon-counting detector. ATLAS operates by gauging the time delay of laser pulses, which last about 1.6 nanoseconds and are emitted at a brisk 10 kHz rate [2]. This results in laser footprints spaced about 0.7 m apart on the satellite's track, each covering roughly 17 m in diameter on the ground. The six parallel ground tracks of ATLAS, organized in three pairs with a 90 m separation within each and a wider 3000 m gap between pairs, provide dense surface coverage [3]. Given that glacial lakes are the result of glacier retreat and melting, and they are situated close to glaciers [4], we utilize ICESat-2's diverse data products—especially ATL06 for land ice and ATL13 for inland water bodies—to precisely measure glacial lake water levels (GLWLs).

The Sentinel-3 satellite constellation, a part of the European Space Agency's Copernicus Programme, offers invaluable radar altimetry data. This series, encompassing Sentinel-3A (launched February 2016) and Sentinel-3B (launched April 2018), operates in a sun-synchronous low earth orbit, repeating its topography package every 27 days with a 4-day sub-cycle [5]. Each satellite is equipped with a dual-frequency synthetic aperture radar altimeter, capable of operating in both high-resolution (300 m) SAR mode and low-resolution (1640 m) LRM mode [5]. The SAR mode, primarily active between 60° N and 60° S, is optimized for high-resolution measurements over varied surfaces, thereby enhancing the accuracy in applications like coastal water analysis and inland water evaluation. Our research leverages Level-2 data from both Sentinel-3A and Sentinel-3B [6].

In sum, the combination of high sampling rates and low revisit characteristics of ICESat-2 with the high revisit rates and low sampling frequencies of Sentinel-3 enables us to extract accurate elevations of glacial lake surfaces, augmenting our understanding of these dynamic components of the cryosphere.

**Glacial Lake Inventory Data**

Considering the launch timeline of ICESat-2 and Sentinel-3 satellites, which occurred around 2018, it is advisable to select a high-quality glacial lake inventory dated around that year, covering the entirety of HMA region. To this end, we utilized the 2018 glacial lake inventory by Wang et al. [7], which specifically focuses on the High-Mountain Asia region. This inventory, based on 2018 Landsat OLI false-color composite imagery, employs visual interpretation methods to delineate glacial lake boundaries with high accuracy. It provides an extensive and reliable dataset for the demarcation of glacial lakes across HMA, aligning seamlessly with the temporal and spatial parameters of altimetry data. The 2018 inventory of glacial lakes in HMA region reveals a substantial total count of 25,385 lakes, covering an extensive area of 1,746.49 km². However, a closer analysis indicates that these lakes are generally small in size. The average area of these lakes is 0.069 km², with a median of 0.023 km². In terms of dimensions, the lakes display shorter length, with mean and median lengths of 0.326 km and 0.225 km, respectively. Their widths are also relatively narrow, averaging 0.179 km and a median of 0.136 km. These dimensions and distributions are further illustrated in Fig. S1.

**Other Ancillary Data**

In addition, to explain the potential causes of glacial lake level changes, this study also utilized the Randolph Glacier Inventory 6.0 [8] product for the parameter of glacier area in the watershed of glacial lake. The NASA DEM provided parameters such as terrain slopes around the glacial lakes and watershed boundary, while the precipitation products offered by the ERA-5 Land reanalysis dataset were used to analyze its intra-annual amplitude.

# Supplementary Methods

**Extracting glacial lake levels from altimetry data**

The extraction of GLWLs from ICESat-2's ATL06 and ATL13 products primarily involves retrieving longitude, latitude, and elevation values. The satellite's high along-track sampling resolution, complemented by its six rows of laser points per trajectory, allows for a dense collection of altimetry points, thereby enhancing the accuracy in capturing the glacial lake level. Following the approach of Zhang et al. [9], we employ the 1.5 NMAD (normalized median absolute deviation) filtering technique to eliminate data outliers at each time point. The remaining laser points are then averaged to determine the water level of the glacial lake for each respective time point.

For extracting glacial lake level values from Sentinel-3 data, we predominantly adopt Xu's method [10]. This method begins with extracting key variable values from the raw data and then applying the following equation to calculate the lake surface level:

$H=H_{\mathrm{alt}}-H_{\mathrm{range}}-H_{\mathrm{geo}}-H_{\mathrm{geoid}}$ (1)

where, $H_{\mathrm{alt}}$ represents the satellite altitude, $H_{\mathrm{range}}$ is the distance between the sensor and the lake surface, $H_{\mathrm{geo}}$ accounts for various geophysical corrections (including ionospheric, wet and dry tropospheric effects, solid earth and ocean loading tides, pole tide, inverse barometer, sea state bias, and high-frequency fluctuations), and $H_{\mathrm{geoid}}$ is the geoid height relative to the WGS84 ellipsoid. Considering Sentinel-3's lower along-track resolution (300m) and the relatively small size of the glacial lakes (with mean and median lengths of 0.326 km and 0.225 km, and widths of 0.179 km and 0.136 km, respectively), only a limited number of radar-measured points fall within the glacial lake surface. Given this constraint, we opted not to apply 1.5 NMAD outlier detection processing. We directly compute the mean value of the radar-measured points at each time point, establishing it as the water level of the glacial lake for that specific time.

**Indirect validation of ICESat-2 and Sentinel-3 in glacial lake levels**

In the rigorous and inaccessible glacial environments where glacial lakes are situated, acquiring long-term in-situ measurements of water levels remains a formidable challenge. Guangxie Co [11], the sole glacial lake in HMA region with extensive in-situ water level data, unfortunately lacks coverage by ICESat-2 and Sentinel-3 satellites. Consequently, there are currently no in-situ level available to validate the accuracy of ICESat-2's laser altimetry and Sentinel-3’s radar altimetry in monitoring GLWLs. However, the accuracy and precision of measuring ground surface water levels using ICESat-2 laser altimetry or Sentinel-3 radar altimetry data have undergone validation against in-situ water level observations. Notably, Cooley et al. [12] have demonstrated the efficacy of ICESat-2 data in water level measurement, comparing it with records from 402 water level gauging stations. This comparison revealed a median standard deviation of 0.017 m and a mean absolute error of 0.14 m. Furthermore, Xu et al. [10] assessed the altimetric performance of ICESat-2, Sentinel-3A, and Sentinel-3B against in-situ water level data from Qinghai Lake, yielding root mean square error values of 0.11 m, 0.07 m, and 0.12 m, respectively. In addition, our study uses an approach for internal validation. When a glacial lake is concurrently monitored by both ICESat-2 and Sentinel-3, with the temporal discrepancy in data acquisition not exceeding seven days, we execute differencing calculations between the water levels as measured by each satellite. This analysis reveals a mean difference of 0.11 m and a median of -0.01 m, as illustrated in Fig. S2a. This internal validation, while indirect, provides a compelling testament to the accuracy of these satellite-based methods in capturing the dynamic water levels of glacial lakes within HMA region.

**Glacial lake level changes based on a periodic fluctuation model**

By integrating ICESat-2 and Sentinel-3 altimetry observations with the 2018 glacial lake mask, our analysis identified a total of 5,943 lakes (23.41%) within HMA that were measured by altimetry satellites more than once. Among these, only 796 lakes (3.14%) were observed at least six times, and 366 lakes (1.44%) received over ten measurements (Fig. S1). Specially, the ICESat-2 laser altimetry satellite observed 5,119 glacial lakes, covering an area of 1,020.796 km², while the Sentinel-3 radar altimetry satellite monitored 1,222 glacial lakes, with a total area of 220.323 km². These findings indicate that a limited number of glacial lakes within HMA have been observed by altimetry satellites. In our study, we analyzed the temporal distribution of water levels in glacial lakes and discerned distinct patterns of both intra-annual amplitudes and inter-annual change rates (Fig. S3c-d). This analysis was further substantiated by the water level records from Guangxie Co, the only glacial lake in HMA with extensive long-term in-situ monitoring data, which aligns with our findings (Fig. S3e).

Given the inherent intra-annual amplitudes in GLWLs, directly applying the annual mean or median values for years with sparse observations to measure inter-annual change rate of glacial lakes levels can lead to misleading interpretations of inter-annual change rates. This is particularly pertinent for ICESat-2, which has a revisit cycle of 91 days. The lake level obtained from ICESat-2 may include a limited number of measurements from periods of peak water levels (typically in July and August) and minimal levels (usually in January and February). Without addressing this intra-annual seasonal bias, subsequent analysis might fail to accurately represent the true nature of the glacial lake level changes over multiple years. Therefore, it's imperative to consider these seasonal changes when examining the inter-annual change rates in GLWLs, especially in cases where the annual dataset is relatively sparse.

To effectively capture both intra-annual amplitude and inter-annual change rate in the behavior of glacial lake water levels, we employ a periodic fluctuation model (Fig. S3a-b and Equation 2) that incorporates both seasonal and long-term change rate components.

$Level\left( t \right)=a\times sin\left( 2\times\pi/T\times t \right)+b\times cos\left( 2\times\pi/T\times t \right)+v\times t+c$ (2)

where t represents the time difference relative to the first water level value, a and b are used to calculate the intra-annual amplitude of the glacial lake level, v represents the inter-annual change rate, and c indicates the mean water level value for the fitted time period. T is the fluctuation period of the glacial lake water level, which is one year (365 days).

The seasonal component, represented by the sine and cosine functions (blue curve in Fig. S3a-b), accounts for intra-annual fluctuations due to the periodic nature of temperature, precipitation, glacier meltwater, evaporation, and other climatic factors that influence glacial lake water level dynamics. The parameters a and b are determined based on observed data, and together, they represent the intra-annual amplitude (Equation 3):

$Amplitude= \sqrt[2]{a^{2}+b^{2}}$ (3)

The inter-annual change rate is captured through a linear term (gray line in Fig. S3a-b), which reflects gradual, long-term net changes in the amount of water in the glacial lake, i.e., the difference between water income and expenditure. This change rate is modeled as a linear function of time, and its magnitude is determined by the coefficient v (Equation 4), which is multiplied by the time t in days, yielding a yearly change rate:

$Change rate=365\times v$ (4)

By combining these two components, the periodic fluctuation model effectively captures both short-term seasonal variations and longer-term change rates, providing a comprehensive framework for analyzing glacial lake dynamics.

The effectiveness of this model in describing both intra-annual and inter-annual variations is demonstrated through its ability to accurately fit glacial lake level data. The model parameters are estimated by fitting the model to in-situ lake level measurements from Guangxie Co, with the resulting seasonal amplitude and long-term change rate matching well with observed lake level fluctuations. In particular, the model’s fit statistics show a higher coefficient of determination (R²) of 0.93, and a lower root mean squared error (RMSE) of 0.06 m. Additionally, similar periodic models have been successfully applied in previous studies of lake water levels [13] and permafrost deformation [14], further supporting the appropriateness of this approach in capturing both intra-annual fluctuations and inter-annual change rates in glacial lake behavior.

The periodic fluctuation model can measure a larger number of glacial lakes, especially those with limited altimetry data, and precisely quantify both intra-annual amplitudes and inter-annual change rates in water levels. However, the model's applicability is constrained for lakes with very sparse (less than six) altimetry data, leading to an analysis on 442 glacial lakes. When fitting these lake water level values using the periodic fluctuation model, we calculated the coefficient of determination (*R*^2^) for each lake, as shown in Fig. S2b. The results revealed a mean of 0.74 and a median of 0.82. This suggests that the intra-annual amplitudes and inter-annual change rates of GLWLs can be quantified using this model.

**Representativeness of the glacial lakes measured in this study**

HMA contains a large number of glacial lakes, but due to limitations in available altimetry data, the number of glacial lakes measured in this study is relatively small.

To comprehensively assess the representativeness of the glacial lakes measured in this study, we compared the probability distributions of all glacial lakes in HMA (25,385 lakes) with those of the 442 glacial lakes measured in this study. This comparison was made across different intervals of area, altitude, longitude, latitude, glacial lake types, annual mean temperature, and total annual precipitation. The values for area, altitude, longitude, latitude, and glacial lake types were derived from the HMA glacial lake inventory provided by Wang et al. [7]. Annual mean temperature and total annual precipitation were calculated based on ERA5-Land data for the period 1990–2023. The detailed results are presented in Fig. S4 below. The glacial lakes in HMA are predominantly small, with 96.49% having an area smaller than 0.3 km². Among the 442 glacial lakes measured in this study, 78.96% have an area smaller than 1 km², and 54.30% have an area smaller than 0.3 km². Therefore, this study includes a significant number of small glacial lakes. Larger glacial lakes (e.g., those with areas > 1 km²) show similar distribution patterns between the two datasets. Specific results are shown in Fig. S4a. Additionally, the probability distributions of glacial lakes in HMA and the measured glacial lakes in this study are similar in terms of altitude (Fig. S4b), longitude (Fig. S4c), latitude (Fig. S4d), annual mean temperature (Fig. S4e), and total annual precipitation (Fig. S4f). Fig. S4g shows that 54.08% of the glacial lakes are classified as ice-uncontacted lakes, 33.47% as non-glacier-fed lakes, 9.44% as ice-contacted lakes, and 3.00% as supraglacial lakes. Among the 442 glacial lakes measured in this study (Fig. S4h), the proportions of these types are 66.97%, 16.06%, 16.29%, and 0.68%, respectively. These proportions align with the statistical results.

In conclusion, the 442 glacial lakes measured in this study provide a good representation of the overall distribution of glacial lakes in HMA in terms of both size and spatial distribution.

**Uncertainty of inter-annual change rate and intra-annual amplitude in glacial lake levels**

To assess the uncertainty of the inter-annual change rates and intra-annual amplitudes of the 442 glacial lakes measured in this study, we employed the 95% upper and lower intervals of each component in the periodic fluctuation model [15], as approximated by the pink and gray shading in Fig. S3b-c. When evaluating the uncertainty of the mean and median inter-annual change rates and intra-annual amplitudes within each mountain range, we utilized the bootstrap method [16]. This approach not only avoids the need for distribution assumptions regarding the inter-annual change rates and intra-annual amplitudes of glacial lakes within each mountain range but also addresses the issue of inconsistent or limited numbers of measured glacial lakes in different mountain ranges effectively.

**Analyzing potential factors influencing the inter-annual change rate and of glacial lakes**

Considering that changes in the glacial lake levels are primarily governed by external hydrological inputs and outputs, this study further concentrated on an in-depth analysis of these processes to elucidate the potential factors contributing to the observed inter-annual change rates. We categorized each glacial lake as either closed or open, based on the presence of a breach or evidence of flowing water, as determined visually through expert visual interpretation [17] of multispectral Sentinel-2 images, supplemented with Google Earth imagery [18, 19]. Moreover, given the fundamental influence of glacier mass balance on the water levels of glacial lakes, we further sought a viable proxy to quantify this relationship due to the unavailability of direct mass balance measurements for the corresponding time. We therefore established a statistical correlation between the change in each glacial lake level and the glacier area in its corresponding watershed (Fig. S3c-d), using glacier area as an indirect indicator.

**Analyzing potential factors influencing the intra-annual amplitude of glacial lakes**

Precipitation, particularly within-year precipitation, directly determines the magnitude of short-term changes in GLWLs. Hence, this study also employs the periodic fluctuation model to calculate the intra-annual amplitudes of precipitation from ERA-5 Land precipitation data, thereby establishing a statistical relationship between within-year precipitation and the intra-annual amplitudes of glacial lake level.

Additionally, the within-year variation in glacial lake water volume often results in the lake area changing from $A_{start}$ to $A_{end}$ and the water level changing from $H_{start}$ to $H_{end}$. Assuming the glacial lake bathymetry has a pyramidal shape [20-23], the relationship between changes in lake volume, water level, and area can be simplified to the following Equation (5). In terms of intra-annual amplitude, the glacial lake area increases from $A_{start}$ to $A_{end}$. According to Equation (5), it can be deduced that with a constant $\Delta v$ and $A_{start}$, the steeper the slope around the glacial lake, the smaller the increase in $A_{end}$. Consequently, this yields a more pronounced change in the water level difference, $H_{start}-H_{end}$. Hence, we suggest that the average slope around the glacial lake may influence the intra-annual amplitudes of the glacial lake level, and, in turn, we establish a statistical relationship between the two.

$\Delta v=(H_{start}-H_{end})\times(A_{start}+A_{end}+\sqrt{A_{start}\times A_{end}})/3$ (5)

Where $\Delta v$ is the volume variation and $H_{start}$, $A_{start}$, and $H_{end}$, $A_{end}$ are the level and area in the start date and end date, respectively.

# Data availability

ICESat-2 laser altimetry data and NASA DEM were obtained from NASA (<https://search.earthdata.nasa.gov/>). Sentinel-2 multispectral image and Sentinel-3 radar altimetry data were from Copernicus Programme of European Space Agency (<https://scihub.copernicus.eu/dhus/#/home>). The temperature and total precipitation of ERA5-Land was download from Copernicus (<https://cds.climate.copernicus.eu/cdsapp#!/home>). The glacier boundaries were obtained from <http://www.glims.org/RGI/rgi60_dl.html>. The 2018 glacial lake inventory of High-Mountain Asian can be downloaded from the link (<https://doi.org/10.12072/casnw.064.2019.db>). The in-situ water level measurements of the Guangxie Co glacial lake are provided by the National Cryosphere Desert Data Center (<http://www.ncdc.ac.cn>).

# Supplementary Figures


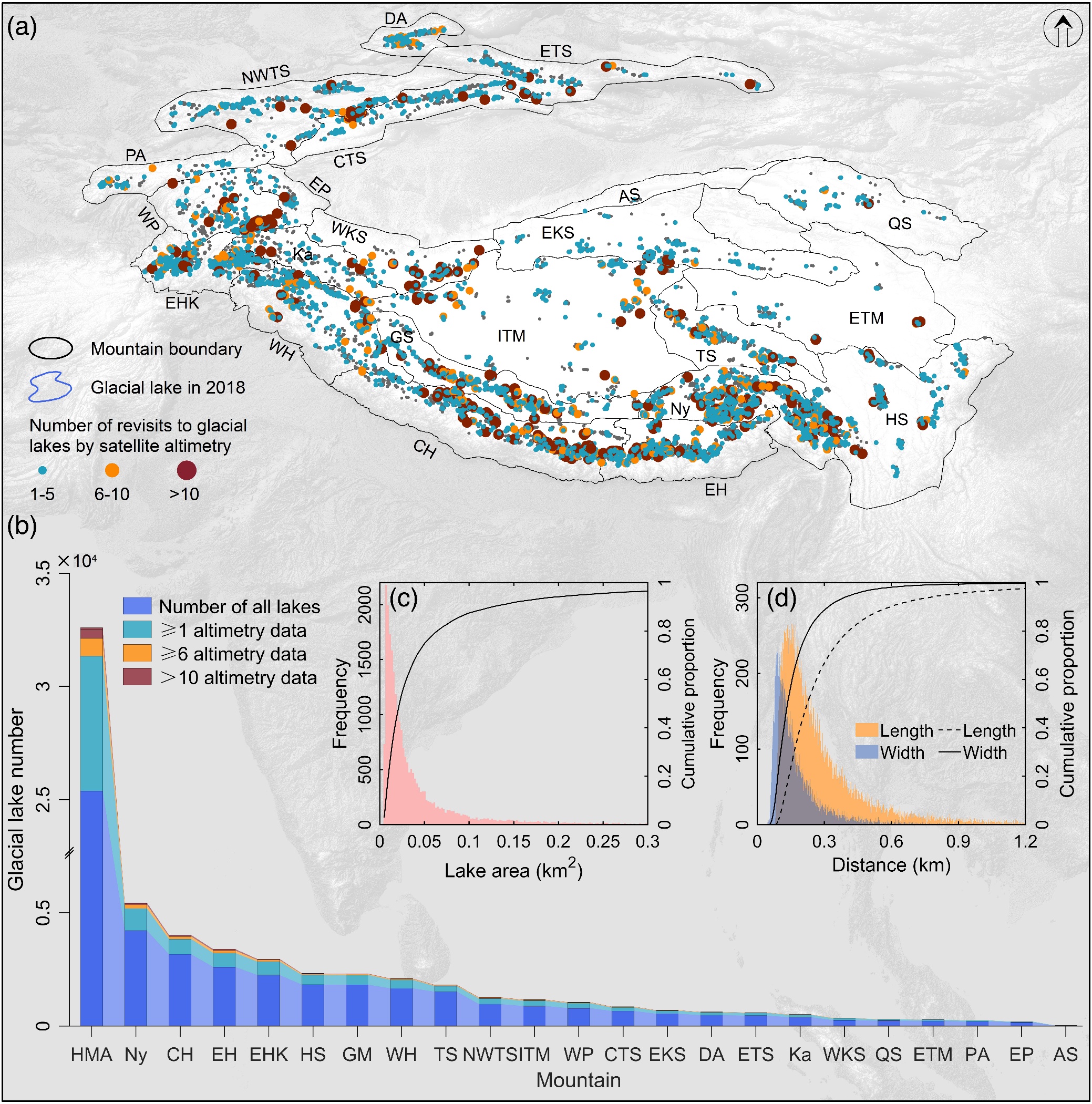


**Supplementary Figure S1 a,** distribution of glacial lakes in HMA and number of measurements of each glacial lake levels by altimetry satellites (ICESat-2 and Sentinel-3). The small steel-blue color circle represents a glacial lake monitored 1‒5 times by altimetry satellites. The moderate orange-red color circle denotes a lake monitored 5‒10 times, and the large maroon color circle signifies a lake observed more than 10 times. **b,** the total number of glacial lakes within each mountain range and the count of glacial lakes monitored at varying frequencies by measurement satellites. **c,** the frequency, and cumulative proportion of glacial lake areas across various size intervals. **d,** the frequency distribution and cumulative proportion of the length and width of the smallest outer rectangle of the glacial lake, categorized into different distance intervals. The background image is derived from NASA DEM, while glacial lake data is sourced from Wang et al.'s inventory [7]. HMA, High Mountain Asia; CH, Central Himalaya; CTS, Central Tien Shan; DA, Dzhungarian Alatau; EH, Eastern Himalaya; EHK, Eastern Hindu Kush; EKS, Eastern Kunlun Shan; EP, Eastern Pamir; ETM, Eastern Tibetan mountains; ETS, Eastern Tien Shan; GM, Gangdise mountains; HS, Hengduan Shan; Ka, Karakoram; NWTS, Northen-Western Tien Shan; Ny, Nyainqêntanglha; QS, Qilian Shan; TS, Tanggula Shan; ITM, Interior Tibetan Mountains; WH, Western Himalaya; WKS, Western Kunlun Shan; WP, Western Pamir.


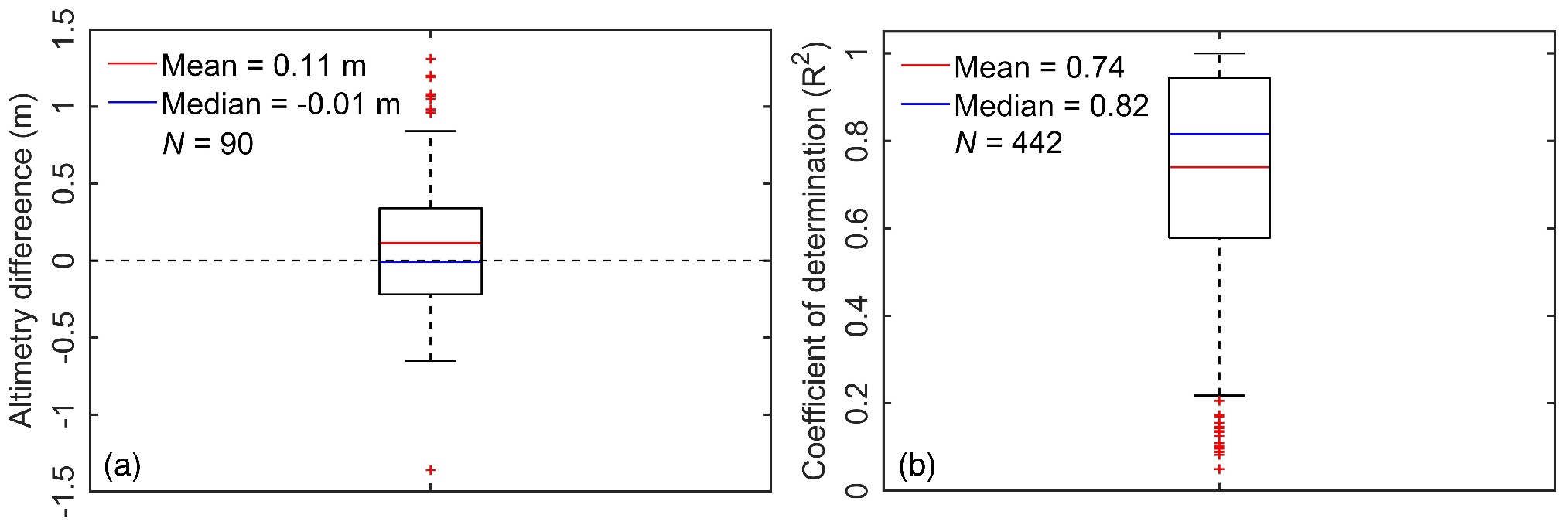


**Supplementary Figure S2 a,** the differences in lake water levels derived from ICESat-2 laser altimetry and Sentinel-3 radar altimetry within a 7-day measurement period. The red line represents the mean value (0.11 m) of the water level differences, while the blue line represents the median value (-0.01 m). **b,** the coefficients of determination (*R^2^*) over time for 442 lake water level measurements fitted with a periodic fluctuation model. The red line represents the mean value (0.74) of the *R^2^*, while the blue line represents the median value (0.82).


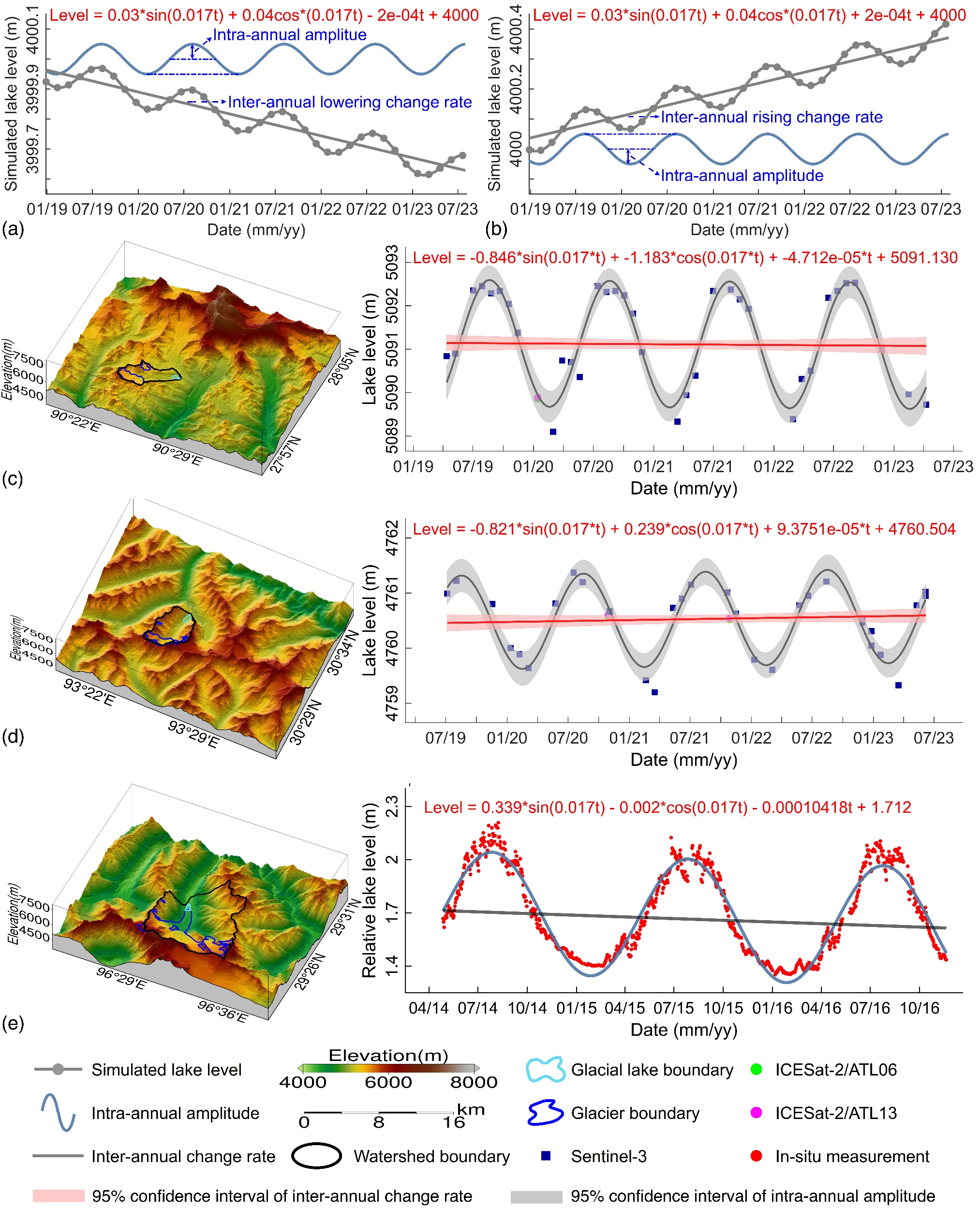


**Supplementary Figure S3** Results of GLWLs measured by altimetry satellites and the application of a periodic fluctuation model to fit both intra-annual amplitudes and inter-annual change rates are presented. **a, b,** Schematic diagrams illustrating the intra-annual amplitude and inter-annual change rates in GLWLs as measured by the periodic fluctuation model based on the simulated glacial lake levels, where **a** shows the inter-annual lowering change rate in glacial lake levels and **b** shows the inter-annual rising change rate in glacial lake levels. **c, d,** Spatial location of two glacial lakes, the parent glacier, the watershed region, and water level values between 2019 and 2023 obtained by ICESat-2 and Sentinel-3 altimetry satellite. The red equation is derived by fitting the water level with a periodic fluctuation model. The pink and gray shaded areas represent the 95% confidence intervals for the fitted curves of the inter-annual change rate and intra-annual fluctuations of GLWLs, respectively. **e,** Spatial location of the Guangxie Co glacial lake, the parent glacier, the watershed region, and relative water level values between April 27, 2014, and November 20, 2016, obtained by pressure transducer. The red equation is derived by fitting the relative water level of Guangxie Co with a periodic fluctuation model.


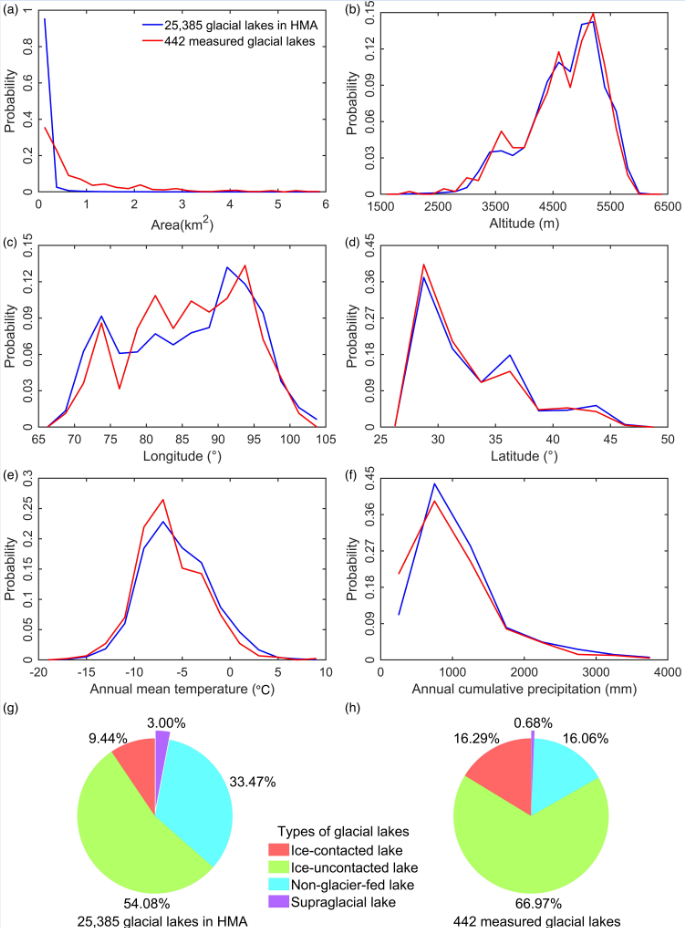


## Supplementary Figure S4 Probability distribution of all (25,385) glacial lakes and measured (442) lakes in different ranges of area (a), elevation (b), longitude (c), latitude (d), mean annual temperature from 1990 to 2023 (e), and total annual precipitation from 1990 to 2023 (f), and proportion of different types of glacial lakes (g, h).


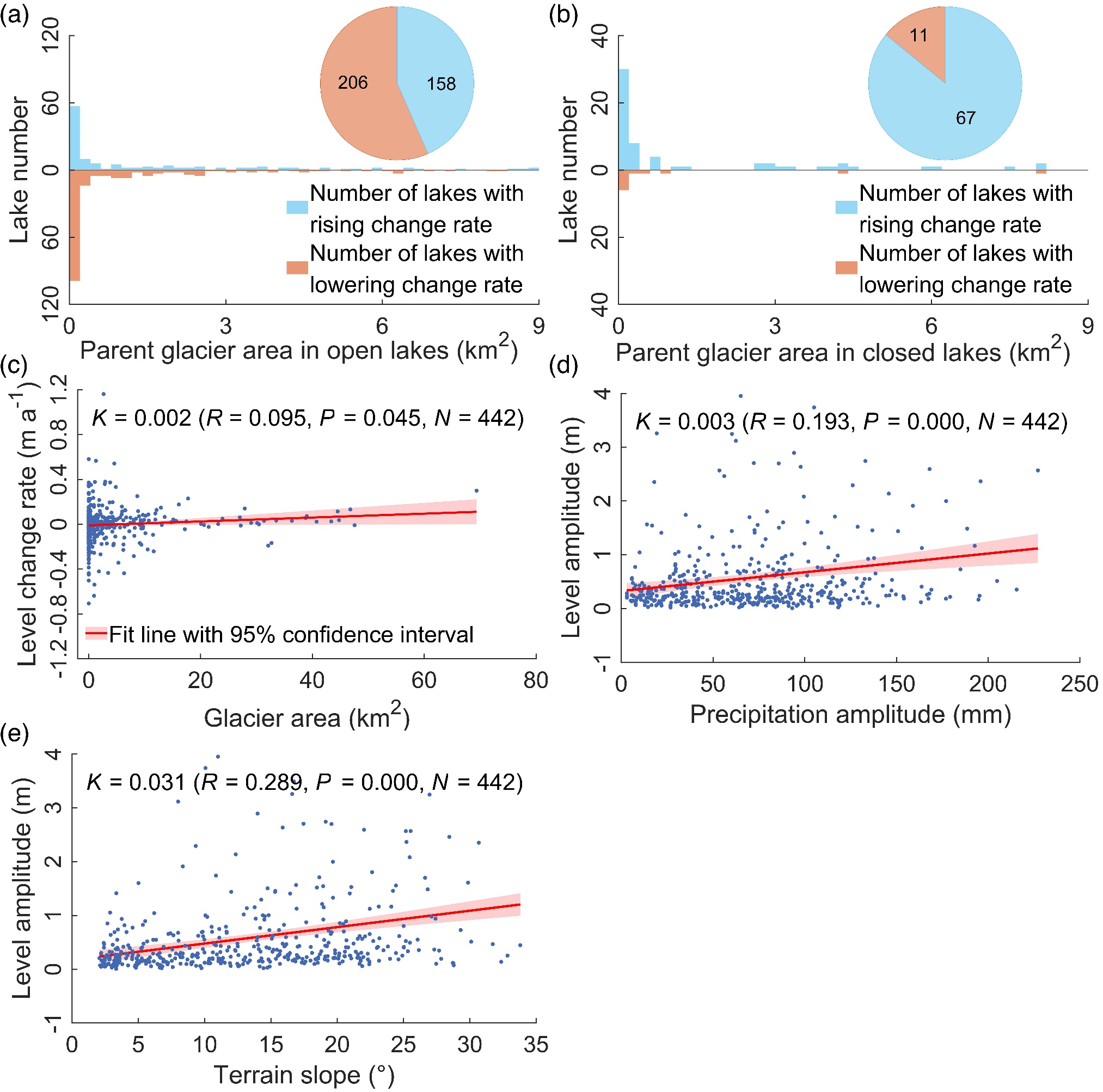


**Supplementary Figure S5** Factors influencing inter-annual change rates and intra-annual amplitudes of water levels for open (a) and for closed (b) glacial lakes. The number of lakes exhibiting a rising or lowering in inter-annual water level varies with the size of glaciers; In panels (a) and (b) the dark-salmon and light-blue colors represent the number of glacial lakes with inter-annual water level lowering and rising, respectively. Panel (c) depicts a linear fit between glacier area and the inter-annual change rates in the water levels of these lakes. Panel (d) depicts a linear fit between intra-annual amplitude of glacial lake levels and concurrent precipitation amplitude. Panel (e) depicts a linear fit between intra-annual amplitude of glacial lakes levels and terrain slope. In panels (c), (d), and (e), all linear relationships include the 95% confidence interval (pink shaded area), and report the slope K, correlation coefficient R, P value, and the number N of data points used for the regression fitting.

# Supplementary Table

## Supplementary Table 1 Reported glacial lake outburst floods (GLOFs) over High Mountain Asia

| ID | Lake name | Outburst date | Mountain | Latitude (°) | Longitude (°) | References |
| --- | --- | --- | --- | --- | --- | --- |
| 1 | Machhapuchhre | 450 years ago | Central Himalaya | 28.515 | 83.992 | Bajracharya et al., 2008 [24]  Zheng et al., 2021 [25] |
| 2 | Chubda Tsho | Before 1956 | Eastern Himalaya | 28.02 | 90.71 | Komori et al., 2012 [26]  Zheng et al., 2021 [25] |
| 3 | Tarikha Lake | Before 1956 | Eastern Himalaya | 28.028 | 90.673 | Komori et al., 2012 [26]  Zheng et al., 2021 [25] |
| 4 | Degaco | Before 1966 | Eastern Himalaya | 28.33 | 90.67 | Komori et al., 2012 [26]  Zheng et al., 2021 [25] |
| 5 | Jhomohari South | Before 1966 | Eastern Himalaya | 27.79 | 89.27 | Komori et al., 2012 [26]  Zheng et al., 2021 [25] |
| 6 | Jichudrake North 1st | Before 1966 | Eastern Himalaya | 27.88 | 89.31 | Komori et al., 2012 [26]  Zheng et al., 2021 [25] |
| 7 | Jichudrake North 2nd | Before 1966 | Eastern Himalaya | 27.88 | 89.35 | Komori et al., 2012 [26]  Zheng et al., 2021 [25] |
| 8 | Simdong Goi Tsho | Before 1966 | Eastern Himalaya | 28.22 | 89.81 | Komori et al., 2012 [26]  Zheng et al., 2021 [25] |
| 9 | Unknown | Before 1966 | Eastern Himalaya | 28.04 | 89.89 | Zheng et al., 2021 [25] |
| 10 | Upper Chokham Tsho | Before 1966 | Eastern Himalaya | 27.84 | 89.37 | Komori et al., 2012 [26]  Zheng et al., 2021 [25] |
| 11 | Upper Jiejiu Tsho | Before 1966 | Eastern Himalaya | 28.27 | 90.71 | Komori et al., 2012 [26]  Zheng et al., 2021 [25] |
| 12 | Upper Shegong Tsho | Before 1966 | Eastern Himalaya | 28.30 | 90.74 | Komori et al., 2012 [26]  Zheng et al., 2021 [25] |
| 13 | Barun Khola East |  | Central Himalaya | 27.829 | 87.095 | Bajracharya et al., 2008 [24] |
| 14 | Barun Khola West |  | Central Himalaya | 27.844 | 87.082 | Bajracharya et al., 2008 [24] |
| 15 | Chokarma Cho |  | Central Himalaya | 27.906 | 86.913 | Bajracharya et al., 2008 [24] |
| 16 | Huang Tsho |  | Eastern Himalaya | 28.266 | 90.069 | Komori et al., 2012 [26] |
| 17 | Unnamed 1st |  | Central Himalaya | 29.221 | 83.703 | Bajracharya et al., 2008 [24] |
| 18 | Unnamed 2nd |  | Central Himalaya | 29.118 | 83.739 | Bajracharya et al., 2008 [24] |
| 19 | Unnamed 3rd |  | Central Himalaya | 29.65 | 82.80 | Bajracharya et al., 2008 [24] |
| 20 | Taraco; Tara-Cho | 1935-08-28 | Central Himalaya | 28.30 | 86.13 | Xu et al., 1989 [27]  Bajracharya et al., 2008 [24]  Wang et al., 2012 [28]  Yao et al., 2014 [29]  Liu et al., 2014 [30]  Nie et al., 2018 [31]  Veh et al., 2019 [32]  Zheng et al., 2021 [25] |
|  |  | 1964-07 |  |  |  | Zheng et al., 2021 [25] |
| 21 | Qubiximaco; Qiongbihema Tsho | 1940-07-10 | Eastern Himalaya | 27.85 | 88.92 | Wang et al., 2012 [28]  Liu et al., 2014 [30]  Veh et al., 2019 [32]  Zheng et al., 2021 [25] |
|  |  | 1970-07 |  |  |  | Zheng et al., 2021 [25] |
| 22 | Lureco | 1950s | Eastern Himalaya | 28.27 | 90.59 | Veh et al., 2019 [32]  Zheng et al., 2021 [25] |
| 23 | Sangwang Tsho | 1954-07-16 | Eastern Himalaya | 28.24 | 90.11 | Bajracharya et al., 2008 [24]  Wang et al., 2012 [28]  Liu et al., 2014 [30]  Veh et al., 2019 [32]  Zheng et al., 2021 [25] |
| 24 | Cuoalong Glacier | 1955-1966 | Eastern Himalaya | 28.06 | 90.61 | Komori et al., 2012 [26]  Veh et al., 2019 [32] |
| 25 | Tarina Tsho | 1957 | Eastern Himalaya | 28.11 | 89.90 | Bajracharya et al., 2008 [24]  Komori et al., 2012 [26]  Veh et al., 2019 [32] |
|  |  | 1959 |  |  |  | Zheng et al., 2021 [25] |
| 26 | Glacial lake(s) in the upstream of Aksay Valley | 1960-07-08 | Northen-Western Tien Shan | 42.53 | 74.54 | Zheng et al., 2021 [25] |
|  |  | 1961-07 |  |  |  |  |
|  |  | 1965-07-21 |  |  |  |  |
|  |  | 1966-06-18 |  |  |  |  |
|  |  | 1968-08-10 |  |  |  |  |
|  |  | 1969-07-25 |  |  |  |  |
|  |  | 1970-07-18 |  |  |  |  |
|  |  | 1970-08-02 |  |  |  |  |
|  |  | 1980-07-18 |  |  |  |  |
|  |  | 1980-08-03 |  |  |  |  |
| 27 | Bachamancha | 1960s | Eastern Himalaya | 28.03 | 90.68 | Veh et al., 2019 [32] |
| 28 | A glacial lake on Lunana Glacier | 1960s | Eastern Himalaya | 28.04 | 90.31 | Nie et al., 2018 [31]  Zheng et al., 2021 [25] |
| 29 | Zhangzangbo;  Cirenmaco 1st | 1964 | Central Himalaya | 28.07 | 86.07 | Veh et al., 2019 [32] |
| 30 | Longda Tsho | 1964-08-25 | Central Himalaya | 28.62 | 85.35 | Bajracharya et al., 2008 [24]  Liu et al., 2014 [30]  Veh et al., 2019 [32] |
|  |  | 1968-08 |  |  |  | Zheng et al., 2021 [25] |
| 31 | Gelhaipuco  Jilaico | 1964-09-21 | Central Himalaya | 27.96 | 87.81 | Wang et al., 2012 [28]  Liu et al., 2014 [30]  Veh et al., 2019 [32] |
| 32 | Damenhaico | 1964-09-26 | Nyainqêntanglha | 29.87 | 93.04 | Veh et al., 2019 [32]  Zheng et al., 2021 [25] |
|  |  | 1968-08 |  |  |  | Zheng et al., 2021 [25] |
| 33 | Name Unknown | 1966 to 1974 | Eastern Himalaya | 27.82 | 89.35 | Veh et al., 2019 [32] |
| 34 | Aya Co | 1965-08 | Central Himalaya | 28.35 | 86.49 | Zheng et al., 2021 [25] |
|  |  | 1968-08-15 |  |  |  | Xu et al., 1989 [27]  Bajracharya et al., 2008 [24]  Liu et al., 2014 [30]  Nie et al., 2018 [31]  Veh et al., 2019 [32]  Zheng et al., 2021 [25] |
|  |  | 1969-08-17 |  |  |  |  |
|  |  | 1970-08-18 |  |  |  |  |
| 35 | Pogeco | 1972-07-23 | Tanggula Shan | 31.74 | 94.73 | Xu et al., 1989 [27]  Yao et al., 2014 [29]  Veh et al., 2019 [32] |
| 36 | Bogeco | 1974-07-06 | Tanggula Shan | 31.86 | 94.76 | Yao et al., 2014 [29]  Veh et al., 2019 [32] |
| 37 | Tuyuksu Glacial Lake | 1973-07-15 | Northen-Western Tien Shan | 43.07 | 77.08 | Zheng et al., 2021 [25] |
| 38 | Angy-Say Glacial Lake | 1974-06-14 | Northen-Western Tien Shan | 41.97 | 77.14 | Zheng et al., 2021 [25] |
|  |  | 1976-06-17 |  |  |  |  |
|  |  | 1980-06-25 |  |  |  |  |
|  |  | 1980-07-14 |  |  |  |  |
| 39 | Nare Lake | 1977-09-03 | Central Himalaya | 27.83 | 86.83 | Bajracharya et al., 2008 [24]  Wang et al., 2012 [28]  Veh et al., 2019 [32] |
| 40 | Nagma Pokhari | 1980-06-23 | Central Himalaya | 27.87 | 87.87 | Bajracharya et al., 2008 [24]  Wang et al., 2012 [28]  Veh et al., 2019 [32] |
| 41 | No. 35 Glacial Lake | 1980-07-23 | Northen-Western Tien Shan | 42.92 | 76.60 | Zheng et al., 2021 [25] |
| 42 | A glacial lake in the upstream of Adygene Valley | 1910 | Northen-Western Tien Shan | 42.54 | 74.42 | Zheng et al., 2021 [25] |
|  |  | 1934 |  |  |  |  |
|  |  | 1953 |  |  |  |  |
|  |  | 1966 |  |  |  |  |
|  |  | 1980 |  |  |  |  |
|  |  | 1982 |  |  |  |  |
|  |  | 1988 |  |  |  |  |
|  |  | 1993 |  |  |  |  |
|  |  | 2012 |  |  |  |  |
| 43 | Zharico | 1981-06-24 | Eastern Himalaya | 28.30 | 90.61 | Xu et al., 1989 [27]  Bajracharya et al., 2008 [24]  Wang et al., 2012 [28]  Yao et al., 2014 [29]  Veh et al., 2019 [32] |
| 44 | Zhangzangbo/  Cirenmaco 2nd | 1981-07-11 | Central Himalaya | 28.07 | 86.07 | Bajracharya et al., 2008 [24]  Liu et al., 2014 [30]  Veh et al., 2019 [32] |
| 45 | Yindapuco | 1982-08-27 | Central Himalaya | 27.95 | 87.91 | Xu et al., 1989 [27]  Bajracharya et al., 2008 [24]  Liu et al., 2014 [30] |
| 46 | Zhangzangbo/  Cirenmaco 3rd | 1983 | Central Himalaya | 28.07 | 86.07 | Veh et al., 2019 [32] |
| 47 | Dig Tsho | 1980-06-28 | Central Himalaya | 27.87 | 86.59 | Zheng et al., 2021 [25] |
|  |  | 1985-08-04 |  |  |  | Bajracharya et al., 2007 [33]  Nie et al., 2018 [31] |
| 48 | Suuktor Glacial Lake | 1985 | Northen-Western Tien Shan | 41.95 | 76.85 | Zheng et al., 2021 [25] |
| 49 |  | 1976-01-04 to 1987-11-08 | Nyainqêntanglha | 30.13 | 93.90 | Veh et al., 2019 [32] |
| 50 | Guangxieco;  Mitui-Cho | 1988-07-15 | Nyainqêntanglha | 29.47 | 96.50 | Bajracharya et al., 2008 [24]  Wang et al., 2012 [28]  Liu et al., 2014 [30]  Veh et al., 2019 [32] |
| 51 |  | 1988-10-09 to 1992-09-25 | Nyainqêntanglha | 29.55 | 92.79 | Veh et al., 2019 [32] |
| 52 |  | 1990-10-20 to 1991-10-23 | Eastern Himalaya | 27.97 | 88.89 | Veh et al., 2019 [32] |
| 53 |  | 1990-11-14 to 1991-09-30 | Eastern Himalaya | 28.09 | 90.33 | Veh et al., 2019 [32] |
| 54 | Chubung | 1991-07-12 | Central Himalaya | 27.88 | 86.47 | Bajracharya et al., 2008 [24]  Wang et al., 2012 [28]  Veh et al., 2019 [32] |
| 55 | Upper Langbu Tsho | 1992-09-22 to  1992-11-01 | Central Himalaya | 27.93 | 86.45 | Nie et al., 2018 [31]  Zheng et al., 2021 [25] |
| 56 |  | 1992-09-04 to 1992-09-20 | Nyainqêntanglha | 29.75 | 96.56 | Veh et al., 2019 [32] |
| 57 |  | 1992-09-23 to 1993-10-28 | Eastern Himalaya | 27.56 | 88.11 | Veh et al., 2019 [32] |
| 58 |  | 1993-08-06 to 1996-10-01 | Eastern Kunlun Shan | 36.03 | 73.2 | Veh et al., 2019 [32] |
| 59 |  | 1993-10-31 to 1994-10-02 | Central Himalaya | 30.34 | 82.14 | Veh et al., 2019 [32] |
| 60 | Zangla Tsho | 1994 | Central Himalaya | 30.36 | 82.12 | Nie et al., 2018 [31] |
| 61 | Luggye Tsho | 1994-10-07 | Eastern Himalaya | 28.09 | 90.30 | Bajracharya et al., 2008 [24] |
|  |  | 2009-04 |  |  |  | Zheng et al., 2021 [25] |
| 62 | Xiaga | 1995-05-26 | Eastern Himalaya | 28.80 | 91.94 | Yao et al., 2014 [29] |
| 63 | Zanaco | 1995-06-06 | Central Himalaya | 28.66 | 85.37 | Bajracharya et al., 2008 [24]  Liu et al., 2014 [30] |
| 64 |  | 1995-11-01 to 1996-10-02 | Central Himalaya | 28.66 | 85.48 | Veh et al., 2019 [32] |
| 65 |  | 1996-10-15 to 1997-10-02 | Eastern Himalaya | 27.7 | 92.39 | Veh et al., 2019 [32] |
| 66 | Kongyangmi La Tsho | 1997-04 to 1997-10 | Eastern Himalaya | 27.90 | 88.78 | Nie et al., 2018 [31]  Veh et al., 2019 [32]  Zheng et al., 2021 [25] |
| 67 |  | 1997-11-01 to 1998-11-04 | Eastern Himalaya | 27.9 | 90.42 | Veh et al., 2019 [32] |
| 68 |  | 1998-11-02 to 1999-08-01 | Central Himalaya | 27.96 | 86.78 | Veh et al., 2019 [32] |
| 69 | Gangri Tsho Ⅲ | Spring-Summer 1998 | Eastern Himalaya | 27.90 | 90.81 | Zheng et al., 2021 [25] |
| 70 | Tam Pokhari/  Sabai Tsho | 1998-09-03 | Central Himalaya | 27.74 | 86.84 | Bajracharya et al., 2008 [24] |
| 71 | Chongbaxia Tsho/  Longjiu Co | 2000-08-06 | Eastern Himalaya | 28.24 | 89.69 | Yao et al., 2014 [29] |
|  |  | 2001 |  | 28.21 | 89.74 | Komori et al., 2012 [26]  Nie et al., 2018 [31]  Veh et al., 2019 [32] |
| 72 |  | 2001-10-24 to 2003-09-28 | Central Himalaya | 28.14 | 85.92 | Veh et al., 2019 [32] |
| 73 | Jialongco 1st | 2002-05-23 | Central Himalaya | 28.21 | 85.85 | Yao et al., 2014 [29]  Liu et al., 2014 [30] |
|  | Jialongco 2nd | 2002-06-29 |  |  |  |  |
| 74 | A glacial lake in the upstream of Shakhdara Valley | 2002-08-07 | Western Pamir | 37.2185 | 71.7345 | Zheng et al., 2021 [25] |
| 75 | Degaco | 2002-09-18 | Eastern Himalaya | 28.33 | 90.67 | Yao et al., 2014 [29] |
| 76 |  | 2002-08-23 to 2004-8-12 | Karakoram | 36.61 | 73.9 | Veh et al., 2019 [32] |
| 77 |  | 2002-10-24 to 2003-10-11 | Nyainqêntanglha | 30.68 | 94.32 | Veh et al., 2019 [32] |
| 78 |  | 2002-11-09 to 2004-10-21 | Nyainqêntanglha | 29.63 | 93.55 | Veh et al., 2019 [32] |
| 79 | Kabache Lake | 2003-08-15 | Central Himalaya | 28.45 | 84.12 | ICIMOD, 2011 [34] |
|  |  | 2004-08-08 |  |  |  |  |
| 80 | Unnamed | 2005 to 2009 | Nyainqêntanglha | 29.75 | 96.47 | Wang et al., 2011 [35]  Veh et al., 2019 [32]  Zheng et al., 2021 [25] |
| 81 | Kashkasuu Glacial Lake | 2006-07-26 to  2006-08-11 | Northen-Western Tien Shan | 41.8571 | 76.9495 | Zheng et al., 2021 [25] |
| 82 | Unknown | 2006-10-07 to  2008-09-26 | Central Himalaya | 27.7926 | 86.8439 | Zheng et al., 2021 [25] |
| 83 | Lang Co | 2007-08-10 | Eastern Himalaya | 27.830 | 91.810 | Yao et al., 2014 [29] |
| 84 |  | 2007-11-21 to 2008-09-20 | Eastern Himalaya | 28.28 | 90.23 | Veh et al., 2019 [32] |
| 85 | Western Zyndan Glacial Lake | 2008-07-24 | Northen-Western Tien Shan | 41.95 | 77.03 | Zheng et al., 2021 [25] |
| 86 |  | 2008-11-12 to 2009-10-14 | Hengduan Shan | 28.32 | 97.84 | Veh et al., 2019 [32] |
| 87 | Tshojo Glacier | 2009-04-29 | Eastern Himalaya | 28.10 | 90.16 | Komori et al., 2012 [26]  Veh et al., 2019 [32] |
| 88 | Zhemai Co | 2009-07-03 | Eastern Himalaya | 28.02 | 92.34 | Yao et al., 2014 [29] |
| 89 | Tsho Ga/Cuoga | 2009-07-29 | Nyainqêntanglha | 30.83 | 94.00 | Yao et al., 2014 [29]  Nie et al., 2018 [31]  Veh et al., 2019 [32] |
| 90 |  | 2009-11-18 to 2010-10-04 | Eastern Himalaya | 28.09 | 90.33 | Veh et al., 2019 [32] |
| 91 | Geiqu | 2010-06-24 to 2010-07-28 | Central Himalaya | 27.95 | 87.99 | Yao et al., 2014 [29]  Nie et al., 2018 [31]  Veh et al., 2019 [32]  Zheng et al., 2021 [25] |
| 92 | Choradari Lake | 2013-06-17 | Central Himalaya | 30.75 | 79.06 | Allen et al., 2015 [36]  Veh et al., 2019 [32] |
| 93 | Ranzeria Co | 2013-07-05 | Nyainqêntanglha | 30.47 | 93.53 | Yao et al., 2014 [29]  Veh et al., 2019 [32] |
| 94 | Jeruy Lake | 2013-08-15 | Northen-Western Tien Shan | 41.99 | 76.81 | Zheng et al., 2021 [25] |
| 95 |  | 2013-10-23 to 2014-11-27 | Nyainqêntanglha | 30.54 | 94.94 | Veh et al., 2019 [32] |
| 96 | Gya Glacier | 2014-07-17 | Northen-Western Tien Shan | 41.97 | 76.83 | Majeed et al., 2021 [37]  Zheng et al., 2021 [25] |
| 97 | Lemthang Tsho | 2015-06-28 | Eastern Himalaya | 28.07 | 89.58 | Veh et al., 2019 [32] |
| 98 | Kargalinka Glacial Lake | 2015-07-23 | Northen-Western Tien Shan | 43.01 | 76.85 | Zheng et al., 2021 [25] |
| 99 | Lhotse Glacier | 2015-05-25 | Central Himalaya | 27.91 | 86.89 | Rounce et al., 2017 [38] |
|  |  | 2016-06-12 |  |  |  |  |
| 100 |  | 2015-09-26 to 2016-09-12 | Central Himalaya | 30.31 | 82.2 | Veh et al., 2019 [32] |
| 101 |  | 2015-10-09 to 2015-10-25 | Central Himalaya | 27.94 | 87.9 | Veh et al., 2019 [32] |
| 102 | Gongbatongsha Tsho | 2016-07-05 | Central Himalaya | 28.08 | 86.06 | Cook et al., 2018 [39]  Nie et al., 2018 [31]  Veh et al., 2019 [32]  Zheng et al., 2021 [25] |
| 103 | Langmale lake | 2017-04-20 | Central Himalaya | 27.81 | 87.14 | Veh et al., 2019 [32]  Byers et al., 2019 [40] |
| 104 | Jinwuco | 2020-06-26 | Nyainqêntanglha | 30.36 | 93.63 | Zheng et al., 2021 [41] |
| 105 | South Lhonak | 2023-10-03 | Eastern Himalaya | 27.9122 | 88.1935 | This study |

# Supplementary References

1. Luo SX, Song CQ, Zhan PF *et al. Catena* 2021; **200**, 105177
2. Markus T, Neumann T, Martino A *et al. Remote Sens Environ* 2017; **190**, 260–273
3. Enderlin EM, Elkin CM, Gendreau M *et al. Remote Sens Environ* 2022; **283**, 113307
4. Taylor C, Robinson TR, Dunning S *et al. Nat Commun* 2023; **14**, 487.
5. Quartly GD, Nencioli F, Raynal M *et al. Remote Sens.* 2020 **12**, 1763
6. Kittel CMM, Jiang LG, Tøttrup C *et al. Hydrol. Earth Syst. Sci.* 2021; **25**, 333–357
7. Wang X, Guo XY, Yang CD *et al.* *Earth Syst Sci Data* 2020; **12**, 2169–2182.
8. RGI Consortium, 2017. Randolph Glacier Inventory - A Dataset of Global Glacier Outlines, Version 6. Boulder, Colorado USA. NSIDC: National Snow and Ice Data Center.
9. Zhang GQ, Chen WF, Xie HJ. *Geophys Res Lett* 2019; **6**, 13107–13118.
10. Xu FL, Zhang GQ, Yi S *et al. J Hydrol* 2022; **604**, 127251.
11. Zhang GQ, Bolch T, Yao TD *et al. Nat Geosci* 2023; **16**, 333–338.
12. Cooley SW, Ryan JC., Smith LC. *Nature* 2021; **591**, 78–81.
13. Ma SM, Liao JJ, Jing RF *et al.* *Big Earth Data* 2024; **8**, 166-188.
14. Li RX, Li ZS, Han JP *et al*. *Int J Appl Earth Obs Geoinf* 2022; **104**, 102527.
15. Zhou YS, Hu J, Li ZW *et al. J Hydrol* 2019; **570**, 38–50.
16. Kulesa A, Krzywinski M, Blainey P *et al. Nat Methods* 2015; **12**, 477–478.
17. Carrivick JL, Quincey DJ. *Glob Planet Change* 2014; **116**, 156–163.
18. Prakash C, Nagarajan R. *Geomat Nat Hazards Risk* 2018; **9**, 337–355.
19. Aggarwal S, Rai SC, Thakur PK *et al. Geomorphology* 2017; **295**, 39–54.
20. Crétaux JF, Abarca-del-Río R, Bergé-Nguyen M *et al. Surv Geophys* 2016; **37**, 269–305.
21. Zhang GQ, Yao TD, Shum CK *et al. Geophys Res Lett* 2017; **44**, 5550–5560.
22. Qiao BJ, Zhu LP, Yang RM, *Remote Sens Environ* 2019; **222**, 232–243.
23. Ke LH, Song CQ, Wang JD *et al.* *Remote Sens Environ* 2022; **268**, 112779.
24. Bajracharya SR, Mool PK, Shrestha BR. *Icfai’s Univ Press India* 2008; 28–46.
25. Zheng GX, Allen SK, Bao A *et al. Nat Clim Change* 2021; **11**, 411–417.
26. Komori J, Koike T, Yamanokuchi T *et al.* *Glob Environ Res* 2012; **16**, 59–70.
27. Xu DM, Feng QH. *Acta Geogr Sin* 1989; **44**, 343–352 (Chinese).
28. Wang X, Liu S, Ding Y *et al.* *Nat Hazard Earth Sys* 2012; **12**, 3109–3122.
29. Yao XJ, Liu SY, Sun MP *et al. J Nat Resour* 2014; **29**, 1377–1390 (Chinese).
30. Liu JJ, Cheng ZL, Su PC*. Quatern Int* 2014; **321**, 78–87.
31. Nie Y, Liu Q, Wang JD *et al.* *Geomorphology* 2018; **308**, 91–106.
32. Veh G, Korup O, Specht SV *et al. Nat Clim Change* 2019; **9**, 379–383.
33. Bajracharya B, Shrestha AB, Rajbhandari L. *Mt Res Dev* 2007; **27**, 336–344.
34. ICIMOD (2011) Glacial lakes and glacial lake outburst floods in Nepal. Kathmandu: ICIMOD
35. Wang WC, Yao TD, Yang XX. *Ann Glaciol* 2011; **52**, 9–17.
36. Allen SK, Rastner P, Arora M *et al. Landslides* 2016; **13**, 1479–1491.
37. Majeed U, Rashid I, Sattar A *et al. Sci Total Environ* 2021; **756**, 144008.
38. Rounce DR, Byers AC, Byers EA *et al. Cryosphere* 2017; **11**, 443–449.
39. Cook KL, Andermann C, Gimbert F *et al. Science* 2018; **362**, 53–57.
40. Byers AC, Rounce DR, Shugar DH *et al. Landslides* 2019; **16**, 533–549.
41. Zheng GX, Mergili M, Emmer A *et al. Cryosphere* 2021; **15**, 3159–3180.
